# Differential Pathogen-Specific Immune Reconstitution in Antiretroviral Therapy-Treated Human Immunodeficiency Virus-Infected Children

**DOI:** 10.1093/infdis/jiy668

**Published:** 2019-01-08

**Authors:** Maximilian Muenchhoff, Emily Adland, Julia Roider, Henrik Kløverpris, Alasdair Leslie, Stephan Boehm, Oliver T Keppler, Thumbi Ndung’u, Philip J R Goulder

**Affiliations:** 1 Department of Paediatrics, University of Oxford, Peter Medawar Building for Pathogen Research, South Parks Road, United Kingdom; 2 HIV Pathogenesis Programme, Doris Duke Medical Research Institute, Nelson R. Mandela School of Medicine, University of KwaZulu-Natal, Durban, South Africa; 3 Max von Pettenkofer Institute, Virology, National Reference Center for Retroviruses, Faculty of Medicine, LMU München, Munich, Germany; 4 German Center for Infection Research (DZIF), partner site Munich, Germany; 5 Department of Infectious Diseases, Ludwig-Maximilians-University, Munich; 6 Africa Health Research Institute (AHRI), Nelson R. Mandela School of Medicine, University of KwaZulu-Natal, Durban, South Africa; 7 Department of Immunology and Microbiology, University of Copenhagen, Denmark; 8 8Department of Infection and Immunity, University College London, United Kingdom; 9 Max Planck Institute for Infection Biology, Berlin, Germany; 10 The Ragon Institute of Massachusetts General Hospital, Massachusetts Institute of Technology and Harvard University, Cambridge

**Keywords:** antiretroviral therapy, cytomegalovirus, HIV, tuberculosis

## Abstract

**Background:**

Susceptibility to coinfections in human immunodeficiency virus (HIV)-infected patients remains increased despite antiretroviral therapy (ART). To elucidate mechanisms involved in immune reconstitution, we studied immune activation, immune exhaustion, and HIV- and copathogen-specific T-cell responses in children before and after ART.

**Methods:**

We prospectively enrolled 25 HIV-infected children to study HIV-, cytomegalovirus (CMV)-, and tuberculosis (TB)-specific T-cell responses before and 1 year after initiation of ART using intracellular cytokine (interleukin-2, interferon-γ, tumor necrosis factor-α) staining assays after in vitro stimulation. We further measured expression of activation, immune exhaustion, and memory phenotype markers and studied proliferative responses after antigen stimulation.

**Results:**

We observed differential, pathogen-specific changes after 1 year of ART in cytokine profiles of CD4 T-cell responses that were associated with shifts in memory phenotype and decreased programmed cell death 1 (PD-1) expression. The proliferative capacity of HIV- and PPD-specific responses increased after 1 year of ART. Of note, the recovery of CMV- and TB-specific responses was correlated with a decrease in PD-1 expression (r = 0.83, *P* = .008 and r = 0.81, *P* = .0007, respectively).

**Conclusions:**

Reconstitution of immune responses on ART is associated with alterations in T-cell phenotype, function, and PD-1 expression that are distinct for HIV, TB, and CMV. The PD-1 pathway represents a potential target for immunotherapy in HIV-infected patients on ART with insufficient immune reconstitution.

## Teaser

Immune activation, immune exhaustion, and T-cell responses were studied in HIV-infected children before and after 1 year of ART. Changes in T-cell functionality were observed that differed by pathogen-specificity and were correlated with alterations of PD-1 expression and memory phenotype.

**(See the Editorial Commentary by Singh and Prasad on pages 1353–5.)**

Untreated chronic human immunodeficiency virus (HIV) infection is characterized by CD4 T-cell depletion, systemic immune activation [1], and immune exhaustion [2, 3] that ultimately result in immunodeficiency. In addition to a quantitative loss of CD4^+^ T cells, cellular immunity in progressive HIV infection is further impaired qualitatively by blunted effector cytokine profiles and reduced proliferative capacity of HIV- [4] and copathogen-specific T cells [5–10] resulting in increased susceptibility to or disease manifestation of copathogens such as cytomegalovirus (CMV) and *Mycobacterium tuberculosis* (MTB).

Although in antiretroviral therapy (ART)-treated adults reconstitution of T-cell responses against MTB [6, 11–13] and CMV [10, 14, 15] appears to be limited, previous studies have shown greater potential for overall immune reconstitution in children on ART [16, 17], which has partly been attributed to increased thymic output [18, 19]. However, most of these studies focused on quantitative immune recovery, whereas data on functional recovery of cellular immunity remain scarce [20–22].

Coinfections with CMV and MTB result in high morbidity and mortality in HIV-infected children especially on the African continent, and therefore it is of great importance to strengthen our knowledge of immune reconstitution against these pathogens in this population. In this prospective longitudinal cohort study, we examine memory differentiation, immune activation, immune exhaustion, and T-cell responses before and 1 year after ART in HIV-infected children compared to HIV-uninfected children. We show that reconstitution of T-cell function on ART differs by pathogen specificity and is associated with shifts in memory phenotype and programmed cell death 1 (PD-1) expression.

## MATERIALS AND METHODS

### Study Subjects

Antiretroviral therapy-naive vertically HIV-infected children and adolescents were recruited at the Ithembalabantu Clinic in Umlazi, Durban, South Africa. Participants were initiated on ART according to current South African guidelines and followed up quarterly for 1 year. Based on sample availability, 25 participants with undetectable viral load levels at the 1-year visit were selected for this study. Three of the participants had a history of tuberculosis (TB) disease and were excluded for the analysis of purified peptide derivative (PPD)-specific immune responses. No participants displayed signs or symptoms of immune reconstitution inflammatory syndrome. In addition, 22 HIV-uninfected siblings (median age, 12.9 years; interquartile range, 8.8–14.95) were studied. Ethical approval for this study was obtained from the University of KwaZulu-Natal Ethics Review Board and the Oxford Research Ethics Committee. For all study participants, written informed consent was given by their caregivers.

### CD4 Count and Viral Load Measurements

Plasma HIV viral load levels were determined using the NucliSens version 2.0 (BioMérieux), and absolute CD4 T-cell counts and percentage (CD4%) were measured by flow cytometry at the Global clinical and viral laboratory (Amanzimtoti, South Africa).

### Cytomegalovirus (CMV) Serology and Quantitative CMV-Polymerase Chain Reaction

Cytomegalovirus serology and polymerase chain reaction testing were performed at the Max von Pettenkofer Institute (LMU München, Munich, Germany).

### Sample Preparation

Peripheral blood mononuclear cells (PBMCs) were isolated from ethylenediaminetetraacetic acid-blood by Ficoll-Hypaque density gradient centrifugation and used directly or cryopreserved in 90% fetal calf serum (FCS) plus 10% dimethyl sulfoxide in liquid nitrogen. Cryopreserved PBMCs were thawed and rested in medium (Roswell Park Memorial Institute 1640 medium [Sigma-Aldrich] plus 10% FCS and 50 units penicillin/streptomycin) for 6 hours before antigen stimulation.

### Antigen Stimulation

The PBMCs were adjusted to 1 million cells/stimulation and stimulated using a pool of 66 peptides covering the HIV-1 Clade C consensus Gag protein (18-mers overlapping by 10 amino acids) at 2 μg/mL final concentration, a pool of 138 peptides covering the CMV pp65 protein (15-mers overlapping by 11 amino acids; NIH AIDS Reagent Program) at 2 μg/mL, MTB PPD (Statens Serum Institute) at 10 μg/mL, and Staphylococcal enterotoxin B (SEB; Sigma-Aldrich) at 1 μg/mL as positive control or medium only. The PBMCs were stimulated overnight (12–16 hours) at 37**°**C in the presence of costimulatory antibodies anti-CD28 and anti-CD49d (BD Bioscience) at 1 μg/mL. After 1-hour incubation, Brefeldin A (Sigma-Aldrich) was added at 10 μg/mL.

### Surface and Intracellular Staining for Flowcytometry

Cell surface and intracellular cytokine staining were performed as previously described [23]. In brief, after cell surface staining, cells were fixed and permeabilized using BD Cytofix/Cytoperm Buffer and stained for intracellular cytokines with antibodies in BD Perm/Wash Buffer (BD Biosciences). Reagents and flowcytometry panels are shown in Supplementary Table 1. Samples were acquired on a BD LSRII and data were analyzed using FlowJo version 10.0.7 (Tree Star). Polyfunctional cytokine profiles were analyzed using Pestle version 1.7 and Spice version 5.35 [24]. Data processing and detailed gating strategies are presented in Supplementary Figures 1–4.

### Carboxyfluorescein Diacetate Succinimidyl Ester Proliferation Assays

Carboxyfluorescein diacetate succinimidyl ester (CFSE)-dilution assays were performed as previously described [25]. Only samples with less than 0.2% CFSE_low_ CD4 and CD8 T cells in the unstimulated control were considered for analysis. Antigen stimulations were performed using the peptide pools and concentrations described above and phytohemagglutinin (PHA) at a final concentration of 2 μg/mL.

### Statistical Analysis

Statistical comparisons between groups were based on Mann-Whitney *U* and Wilcoxon rank-sum tests for unmatched and paired samples, respectively. Spearman rank tests were used for correlations. All of these statistical analyses were performed using GraphPad Prism version 6.0c (GraphPad Software Inc.). Statistical comparisons of multicomponent distributions of polyfunctional profiles as shown in the pie charts were done by permutation tests based on χ^2^ analysis using Spice version 5.35 as previously described [24].

## RESULTS

### Effects of Antiretroviral Therapy on T-Cell Memory Profiles, Activation, and Exhaustion

**Figure 1.**
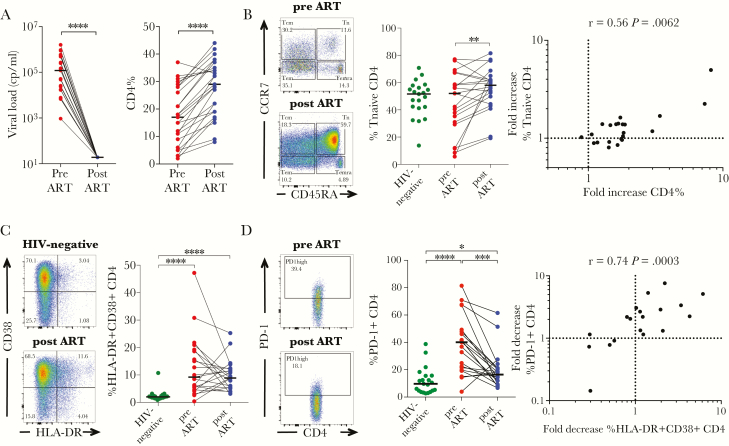
Effect of antiretroviral therapy (ART) on T-cell activation, programmed cell death 1 (PD-1) expression, and memory phenotype. (A) Suppression of viremia and recovery of CD4% after 1 year of ART in the 25 human immunodeficiency virus (HIV)-infected children selected for this study. Medians are shown as horizontal bars. (B) Representative fluorescence-activated cell sorting (FACS) data showing CD45RA and CCR7 expression of CD4 T cells of child 205-33-0015-1 before (top dot plot; CD4 count: 35 cells/mm^3^, CD4%: 2%, viral load: 25910 cp/mL) and after 1 year of ART ([bottom dot plot] CD4 count, 692 cells/mm^3^; CD4%, 16%; viral load, <20 cp/mL). Middle panel shows the frequency of naive (CD45RA^+^CCR7^+^) CD4 T cells in HIV-infected children before and 1 year after ART compared to HIV-uninfected children. The fold increase of the naive CD4 T-cell population (frequency of naive CD4 T cells after 1 year of ART/frequency of naive CD4 T cells before ART) correlates with the fold increase of CD4% (CD4% after 1 year of ART/CD4% before ART). (C) Representative FACS data showing CD38 and HLA-DR expression of CD4 T cells in an HIV-uninfected child (top panel) compared with child 205-33-0061-1 after 1 year of ART ([bottom panel] CD4 count, 511 cells/mm^3^; CD4%, 15%; viral load, <20 cp/mL). Frequencies of activated CD4^+^ T cells (CD38^+^HLA-DR^+^) of HIV-infected children before and after 1 year of ART are shown in comparison with HIV-uninfected children. (D) Representative FACS data for PD-1-expression of CD4 T cells of child 205-33-0061-1 before ([top panel] CD4 count, 251 cells/mm^3^; CD4%, 9%; viral load, 540000 cp/mL) and after 1 year of ART ([bottom panel] CD4 count, 511 cells/mm^3^; CD4%, 15%; viral load, <20 cp/mL). Frequencies of PD-1^+^ CD4 T cells are shown for HIV-infected children before and after ART and in comparison with HIV-uninfected children. Correlation between the fold decrease of PD-1_high_ CD4 T cells (frequency of PD-1^+^ CD4 T cells before ART/frequency of PD-1^+^ CD4 T cells after ART) and activated CD4 T cells after 1 year of ART (frequency of CD38^+^HLA-DR^+^ CD4 T cells before/frequency of CD38^+^HLA-DR^+^ CD4 T cells after ART). Statistical comparisons between groups are based on Mann-Whitney *U* and Wilcoxon rank-sum tests for unmatched and paired samples, respectively (A–D). Spearman rank tests are shown for correlations (B and D) (*, *P* < .05; **, *P* < .01; ***, *P* < .001; ****, *P* < .0001).

To better understand the impact of ART on immune reconstitution at young age, we studied 25 HIV-infected children and adolescents before and 1 year after initiation of ART. Clinical characteristics of study participants are summarized in Table 1 and Supplementary Table 2. Participants were selected based on virologic suppression at the 1-year study visit. The mean increase in absolute CD4 count was 324 (IQR, 121–530) and the mean increase in CD4% was 10.6 (IQR, 5.0–15.2) (Figure 1A). Reconstitution of the CD4 T-cell compartment was associated with an increased frequency of T-naive CD4 T cells (Figure 1B), but not other CD4 memory subsets (data not shown). We also observed an increase in the frequency of naive CD8 T cells (CD45RA^+^CCR7^+^) accompanied by a decrease of central and effector memory CD8 T cells (Supplementary Figure 5a).

**Table 1.** Summary Clinical Characteristics of Study Participants (n = 25)^a^

			Pre-ART			Post-ART		
	Age (Years)	Male/Female	CD4 Count (Cells/mm^3^)	CD4%	Viral Load (Copies/mL)	CD4 Count (Cells/mm^3^)	CD4%	Viral Load (Copies/mL)
Median	7.6	11/24	397	17	120 000	692	29	All <20
IQR	4.5–11.0		183–800	7.5–28	20 798–515 000	494–1033	19–36	

Abbreviations: ART, antiretroviral therapy; IQR, interquartile range.

^a^Median values and IQRs are shown for each clinical parameter before and after 1 year of ART: pre-ART, baseline study visit on the day of ART-initiation; post-ART, study visit 1 year after ART-initiation.

There was no significant decrease in CD4 and CD8 T-cell activation across the cohort but persistently elevated activation levels, characterized by HLA-DR and CD38 expression [26], compared with HIV-uninfected controls after 1 year on therapy (Figure 1C and Supplementary Figure 5b). In the analysis by memory subset, a significant decrease of activation was observed only for CD4 and CD8 central memory T (Tcm) cells (Supplementary Figure 5b).

In contrast, although PD-1 expression was strongly correlated with activation (CD38 and HLA-DR expression) of CD4 T cells at baseline (r = 0.59, *P* = .001; Supplementary Figure 5c), PD-1 expression was significantly reduced in CD4 and CD8 T cells after 1 year of ART (Figure 1D and Supplementary Figure 5d). Because PD-1 expression has been reported to vary between memory subsets [27], we measured PD-1 expression on different subsets to rule out confounding effects of shifts in memory differentiation on ART. The PD-1 expression was reduced across all CD4 subsets, but it persisted at higher levels than in HIV-uninfected controls. A similar pattern was observed for PD-1 expression on CD8 T cells (Supplementary Figure 5d). The decrease of CD4 T-cell PD-1 expression after 1 year of ART was related to the decrease of CD4 T-cell activation (Figure 1D).

### Changes in Functional Profiles and Memory Phenotype of Human Immunodeficiency Virus-Specific CD4 T Cells on Antiretroviral Therapy

**Figure 2.**
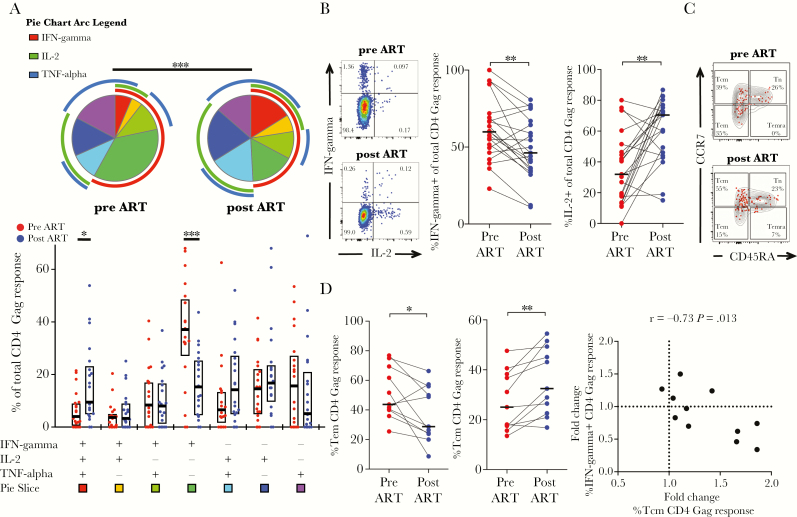
Changes in functional profiles correlate with shifts in memory phenotype of human immunodeficiency virus (HIV)-specific CD4 T cells on antiretroviral therapy (ART). (A) Comparison of HIV-1 Gag-reactive CD4 T cells of 21 children before and after 1 year of ART. Each slice of the pie chart represents the average relative proportions of total Gag-reactive cells producing each possible combination of the cytokines measured. The arcs illustrate the proportions of specific cytokine responses. Interferon (IFN)-γ-monoproducing cells are the predominant Gag-reactive CD4 T cells before ART. Human immunodeficiency virus-specific CD4 T cells from HIV-infected children before ART (red dots) have a qualitatively different functional profile compared with after 1 year of ART (blue dots). The box plots represent the median values and interquartile range of the proportion of the respective functional response toward the total CD4 T-cell response against HIV Gag. (B) Representative fluorescence-activated cell sorting plots showing the frequencies of IFN-γ and interleukin (IL)-2 responding CD4 T cells of child 205-33-0065-1 before ([top] panel] CD4 count, 195 cells/mm^3^; CD4%; 11%, viral load; 250000 cp/mL) and 1 year after ART ([bottom panel] CD4 count, 430 cells/mm^3^; CD4%, 19%; viral load, <20 cp/mL). The percentage of the contribution of the indicated functional response (IFN-γ, left; IL-2, right) toward the total CD4 T-cell response against HIV Gag are shown for HIV-infected children before (red) and after (blue) ART. Decreased proportions of IFN-γ and increased proportions of IL-2 Gag-responding CD4 T cells after ART were observed. (C) Representative dot plots showing the memory maturation profile of the total CD4 population (gray density plot) and of CD4 T cells responding with any cytokine against HIV Gag (Boolean combination of IFN-γ- and/or IL-2- and/or TNF-α-positive CD4 T cells [red dots]). The proportions for each memory subset of the total Gag response are given in the quadrants. Data are shown for child 205-33-0066-1 before ([top panel] CD4 count, 251 cells/mm^3^; CD4%, 9%; viral load, 540 000 cp/mL) and after 1 year of ART ([bottom panel] CD4 count, 511 cells/mm^3^; CD4%, 15%; viral load, <20 cp/mL). (D) The proportions of Gag-responding CD4 T cells with T effector memory (Tem) (CD45RA^−^CCR7^−^) and central memory T (Tcm) (CD45RA^−^CCR7^+^) memory maturation are shown for n = 11 children before (red) and 1 year after ART (blue). The correlation is shown between the fold change of the proportion of Gag-responding CD4 T cells with Tcm phenotype after 1 year of ART (%Tcm of total response after ART/%Tcm response before ART) and the fold change of the proportion of IFN-γ responding cells of the total CD4 Gag response (%IFN-γ responding cells after ART/%IFN-γ responding cells before ART). Increased proportions of Gag-responding CD4 T cells with central memory phenotype (CD45RA^−^CCR7^+^) correlate with decreased IFN-γ responses. Pie charts were compared using permutation tests based on χ^2^ analysis (A). Statistical comparisons between groups are based on Wilcoxon rank-sum tests for paired samples (A, B, and D). Spearman rank test correlation coefficients are shown (D) (*, *P* < .05; **, *P* < .01; ***, *P* < .001).

We next compared cytokine profiles of Gag-specific T cells before and after 1 year of ART. Gag-specific CD4 responses that were detected in 21 subjects at both time points were considered for this comparison. We observed a shift in cytokine patterns for the Gag-specific CD4 T-cells response, with a reduction of interferon (IFN)-γ monoproducing cells (*P* = .001) and increased proportions of cells responding with all 3 cytokines (*P* = .01) (Figure 2A). Although the proportions of cells responding with IFN-γ decreased (*P* = .0025), the fraction of CD4 T cells producing interleukin (IL)-2 upon Gag stimulation increased after 1 year of ART (*P* = .0014) (Figure 2B). Because varying cytokine profiles have been described for different CD4 T-cell memory subsets [28], we hypothesized that these changes in functionality were due to shifts in memory subsets and determined the memory phenotype of cytokine-positive cells in a subset of 11 children (Figure 2C). There was a shift of Gag-specific CD4 T cells from effector memory (*P* = .024) towards central memory phenotype (*P* = .002). This increased proportion of Tcm of the total response was correlated with the decrease of IFN-γ-secreting cells (r = −0.73, *P* = .013) (Figure 2D). Overall, these data show that ART results in changes of memory phenotype of HIV-specific CD4 T-cell responses that is associated with altered functional profiles. Of note, we did not observe significant changes in the magnitude or cytokine profiles of Gag-specific CD8 T cells or in the magnitude of CD4 Gag responses (Supplementary Figure 6a–c).

### Limited Reconstitution of Purified Peptide Derivative-Specific T-Cell Responses on Antiretroviral Therapy

**Figure 3.**
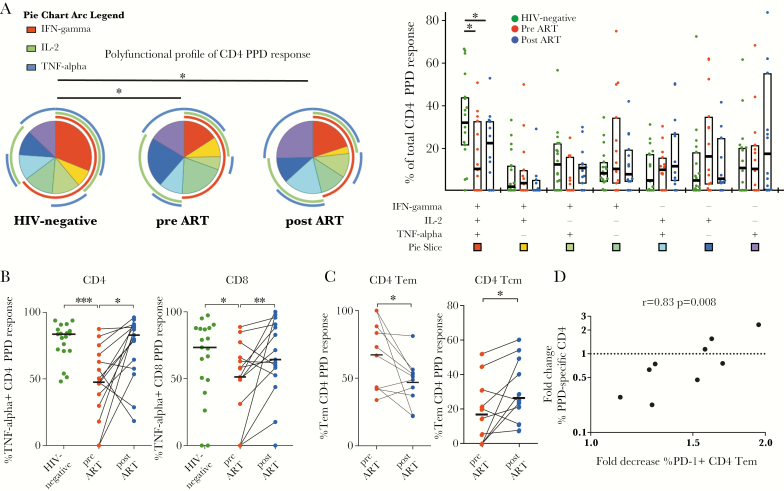
Limited changes in phenotype and function of mycobacteria-specific T-cell responses in human immunodeficiency virus (HIV)-infected children on antiretroviral therapy (ART). (A) Comparison of purified peptide derivative (PPD)-reactive CD4 T cells of 15 children before and after 1 year of ART relative to 22 HIV-uninfected children. Each slice of the pie chart represents the average relative proportions of total PPD-reactive cells producing each possible combination of the cytokines measured. The arcs illustrate the proportions of specific cytokine responses. The PPD-specific CD4 T cells from HIV-infected children before ART (red dots) and after ART (blue dots) show persistently reduced proportions of polyfunctional PPD-reactive CD4 T cells compared with HIV-negative children (green dots). Median values and the interquartile rage are indicated by the bar graphs. (B) Proportions of tumor necrosis factor (TNF)-α-responding CD4 and CD8 T cells are decreased in HIV-infected children before ART (red dots) compared with HIV-negative children (green dots). After 1 year of ART, proportions of TNF-α-responding T cells (blue dots) are restored to levels similar to HIV-negative children. Median values are indicated by the bar graph. (C) Decreased proportions of T effector memory (Tem) CD4 T cells (left panel) and increased proportions of central memory T (Tcm) CD4 T cells of the total PPD-specific CD4 response in children after 1 year of ART (blue dots) compared with before ART (red dots). Data are shown for n = 9 children with available samples for this analysis. (D) Association between the decrease of programmed cell death 1 (PD-1)^+^ CD4 Tem cells on ART (%PD-1^+^ Tem CD4 T cells before ART/%PD-1^+^ Tem after ART) and the change of the total CD4 PPD response (%cytokine-positive CD4 T cells after ART/%cytokine-positive CD4 T cells before ART). Pie charts were compared using permutation tests based on χ^2^ analysis (A). Statistical comparisons between groups are based on Mann-Whitney *U* and Wilcoxon rank-sum tests for unmatched and paired samples, respectively (A–C). Spearman rank test correlation coefficients are shown (D) (*, *P* < .05; **, *P* < .01; ***, *P* < .001).

To better understand the increased susceptibility of HIV-infected children to TB even despite effective ART, we compared PPD-specific T-cell functionality before versus after ART and relative to HIV-uninfected children. Three participants were excluded for this analysis because of TB history. We detected PPD-specific responses in 15 of 22 HIV-infected children and in 19 of 22 HIV-uninfected children. Compared to HIV-uninfected children, HIV-infected children showed reduced frequencies of polyfunctional CD4 T cells responding to PPD with lower proportions of cells producing all 3 Th1 effector cytokines tested (*P* = .0125), and this qualitative alteration persisted after 1 year of ART (*P* = .02) (Figure 3A). In particular, the proportion of cells responding with tumor necrosis factor-α was reduced in CD4^+^ and CD8^+^ T cells of HIV-infected children (*P* = .0002 and *P* = .049, respectively), but it recovered to a certain degree upon ART (*P* = .01 and *P* = .0085) (Figure 3B). Other than this, we observed no significant differences in functional profiles of PPD-responding CD8 T cells between HIV-uninfected children and HIV-infected children before or after ART (Supplementary Figure 7a and b). Similar to our observations for the Gag-specific CD4 response, there was a shift of the PPD response from effector memory phenotype to central memory phenotype (Figure 3C). Of note, although we observed no concordant increase or decrease in the magnitude of the CD4 or CD8 response against PPD (Supplementary Figure 7c), the fold change of the frequency of PPD-responding CD4 T cells after ART was strongly correlated with the decrease in PD-1 expression on CD4 effector memory T cells (r = 0.833, *P* = .008; Figure 3D). It is interesting to note that a similar association was observed for the fold change of the number of PPD-responding CD4 T cells per mm^3^ blood and the change of PD-1 expression on T effector memory (Tem) CD4 T cells after ART (r = 0.85, *P* = .0061; Supplementary Figure 7d), but not for PD-1 expression of the bulk CD4 population or of other CD4 memory subsets. These correlations were still significant after Bonferroni adjustment for multiple comparisons.

### Effects of Antiretroviral Therapy on Cytomegalovirus-Specific T-Cell Responses

**Figure 4.**
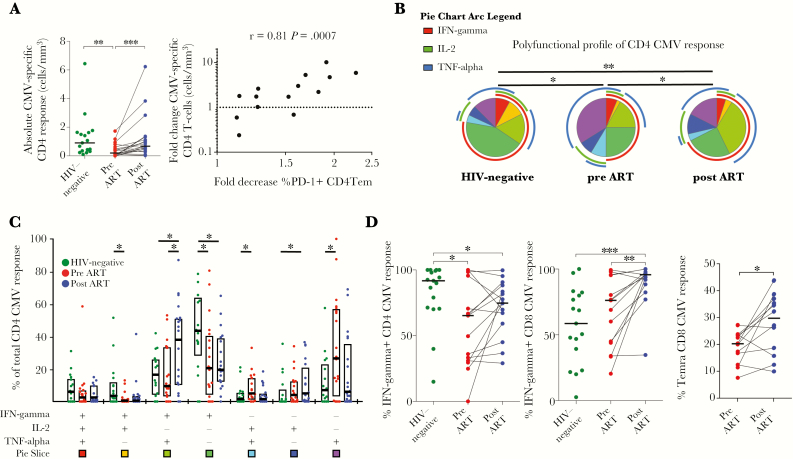
Recovery of cytomegalovirus (CMV)-specific T-cell responses in human immunodeficiency virus (HIV)-infected children on antiretroviral therapy (ART). (A) Comparison of the number of CMV-responding CD4 T cells per mm^3^ of blood in 19 HIV-infected children before (red dots) and after 1 year of ART (blue dots) and relative to 22 HIV-uninfected children (green dots). Horizontal lines represent median values. The fold increase of the numbers of CMV-responding CD4 T cells (CMV-specific CD4 T cells per mm^3^ blood after ART/CMV-specific CD4 T cells per mm^3^ blood before ART) in HIV-infected children after 1 year of ART correlates with decreased frequencies of programmed cell death 1 (PD-1)^+^ CD4 T effector memory (Tem) cells (%PD-1^+^ Tem CD4 T cells before ART/%PD-1^+^ Tem after ART). (B and C) Comparison of cytokine profiles of CMV-reactive CD4 T cells of 15 children with a detectable response before and after 1 year of ART relative to HIV-uninfected children. Each slice of the pie chart represents the average relative proportions of total PPD-reactive cells producing each possible combination of the cytokines measured. The arcs illustrate the proportions of specific cytokine responses. (B) Polyfunctional profiles of CMV-reactive CD4 T cells. Median values and the interquartile rage are indicated by the bar graphs (C). (D, left panel) Decreased proportions of interferon (IFN)-γ-responding CMV-specific CD4 T cells in HIV-infected children before (red dots) and after ART (blue dots) compared with HIV-uninfected children (green dots). (Middle panel) Increased proportions of IFN-γ-responding CD8 T cells in children after 1 year of ART. (Right panel) Increased proportions of CMV-responding CD8 T cells with Temra memory phenotype in children after 1 year on ART. Horizontal bars indicate median values. Pie charts were compared using permutation tests based on χ^2^ analysis (B). Statistical comparisons between groups are based on Mann-Whitney *U* and Wilcoxon rank-sum tests for unmatched and paired samples, respectively (A, C, and D). Spearman rank correlation coefficient and *P* value are shown (A). (*, *P* < .05; **, *P* < .01; ***, *P* < .001).

To further study distinct reconstitution of copathogen immunity, we evaluated CMV-specific responses before and after ART and in comparison to HIV-uninfected children. All children tested seropositive for anti-CMV immunoglobulin (Ig)G. Limited sample availability restricted the analyses of CMV T-cell responses to 19 of the HIV-infected children. We detected CMV-specific CD8 T-cell responses in all HIV-infected and -uninfected children, whereas CD4 T-cell responses against CMV were only detected in 15 of 19 HIV-infected before ART but in all children after 1 year of ART and in the HIV-uninfected controls. It is interesting to note that CMV-deoxyribonucleic acid was only detected in the serum of 4 HIV-infected children before ART at low levels (617, 170, <90, and <90 cp/mL). Two of these children had undetectable CMV-specific CD4 responses, and the other 2 had low numbers of responding cells (lowest quartile) (Figure 4A).

In contrast to the Gag- and PPD-specific CD4 response, for which we observed no increase in the absolute number of antigen-specific cells, the number of CMV-specific CD4 T cells per mm^3^ of blood expanded significantly on ART (*P* = .0005) (Figure 4A). Of note, this increase in absolute counts of CMV-responding CD4 T cells was not merely correlated with higher CD4 counts in children on ART (absolute CMV response vs CD4 count before ART: r = 0.1, *P* = .67; after ART: r = 0.19, *P* = 0.43) nor with the increase of CD4 count (fold change of absolute CMV response vs fold change of CD4 count: r = 0.16, *P* = .58, Spearman rank tests). However, similar to the PPD response, this change in absolute magnitude was strongly correlated with a decrease of PD-1 expression on Tem CD4 T cells (r = 0.81, *P* = .0007), indicating the association between immune exhaustion and reconstitution of copathogen-specific responses on ART (Figure 4A).

The functional profile of CMV-specific CD4 responses varied between HIV-infected and -uninfected children and changed after 1 year of ART (Figure 4B and C). Compared to HIV-uninfected children, the proportion of CMV-specific CD4 T cells responding with IFN-γ was decreased in HIV-infected children (*P* = .029) and remained lower after 1 year of ART (*P* = .051) (Figure 4D). In the CD8 response, the fraction of IFN-γ-producing cells was markedly increased in children on ART compared to before ART (*P* = .0012) and HIV-uninfected children (*P* = .0007) (Figure 4D and Supplementary Figure 8). Although the memory phenotype of CMV-specific CD4 T cells did not change significantly, the proportion of CMV-specific CD8 T cells with Temra memory phenotype (CD45RA^+^CCR7^−^) increased on ART (*P* = .0161) (Figure 4D). It is interesting to note that longitudinal studies of primary CMV infection have shown that CD45RA re-expression of CD8 Tem (CD45RA^−^CCR7^−^) to the CD45RA^+^CCR7^−^ Temra phenotype increased progressively over time and correlated with control of viremia [29].

### Recovery of T-Cell Proliferative Capacity on Antiretroviral Therapy

**Figure 5.**
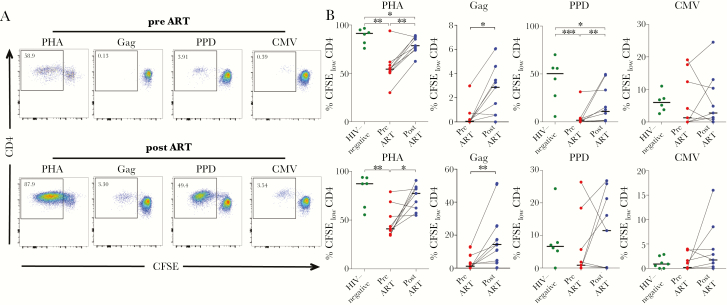
Recovery of T-cell proliferative capacity in human immunodeficiency virus (HIV)-infected children on antiretroviral therapy (ART). (A) Representative fluorescence-activated cell sorting plots for carboxyfluorescein diacetate succinimidyl ester (CFSE)-dilution proliferative responses gated on viable CD4 T cells after 7-day in vitro stimulation with the indicated stimulations in children before and after 1 year of ART. Data are shown for child 205-33-0067-2 before ([top panel] CD4 count, 443 cells/mm^3^; CD4%, 18%; viral load, 1 600 000 cp/mL) and after 1 year of ART ([bottom panel] CD4 count, 631 cells/mm^3^; CD4%, 29%; viral load, <20 cp/mL). (B) Comparison of CFSE_low_ proliferative CD4 and CD8 T-cell responses of HIV-infected children (n = 9) before (red dots) and after ART (blue dots) and relative to HIV-uninfected children (green dots, n = 6). Medians are shown as horizontal bars. Statistical comparisons between groups are based on Mann-Whitney *U* and Wilcoxon rank-sum tests for unmatched and paired samples, respectively (*, *P* < .05; **, *P* < .01; ***, *P* < .001).

We next hypothesized that the reduction in PD-1 expression and change in memory phenotypes of CD4 and CD8 T cells on ART would result in reconstitution of T-cell proliferative capacity. Therefore, we assessed proliferative responses in a CFSE dilution assay before and after ART and compared the responses to HIV-uninfected controls. The proliferative capacity of CD4 and CD8 T cells upon mitogen (PHA) stimulation was significantly reduced in HIV-infected children compared to HIV-uninfected children and recovered partially on ART (Figure 5A and B). The proliferative response of Gag-specific CD4 and CD8 T cells increased after 1 year on ART (Figure 5A and B). It is interesting to note that for the CD4 PPD-specific response, we observed decreased proliferative capacity in HIV-infected children that only incompletely recovered on ART. In contrast, no significant changes were observed for the PPD-specific or CMV-specific responses of CD8 T cells in respect to HIV infection or treatment status (Figure 5A and B).

## DISCUSSION

Antiretroviral therapy potently suppresses viral replication and results in robust quantitative replenishment of CD4 T cells in most HIV-infected children. However, the extent and mechanisms of qualitative recovery of T-cell function remain unclear. In this prospective observational cohort study, we examine the reconstitution of T-cell responses against HIV, CMV, and MTB of 25 HIV-infected children in response to ART. Our main findings were that some of the functional changes of T-cell responses coincided with a reduction of PD-1 expression and shifts in memory phenotype, but that distinct patterns of immune reconstitution were observed for individual pathogens.

In children, de novo production of naive cells by the thymus [18, 30] is believed to allow for greater potential for successful immune reconstitution compared with adults. In adults, the reduced T-cell receptor repertoire due to loss of CD4 T cells during untreated infection may remain truncated if the increase in CD4 count mainly stems from residual cell proliferation and survival [19]. In our study, the recovery of CD4% was strongly correlated with an increase in the frequency of naive CD4 T cells, indicating replenishment of the CD4 compartment by active thymopoiesis at a young age. Apart from age, the degree of immune reconstitution is also influenced by other critical factors such as baseline CD4 count [31], immune activation [26], immune exhaustion [32], and the maturation state of antigen-specific cells [11].

Persistent antigen exposure in combination with the proinflammatory cytokine milieu in untreated HIV infection drives the expansion of effector T-cell populations. Consistent with previous reports in HIV-infected children [21] and a recent cross-sectional cohort study from our group [33], we demonstrate in this study that the HIV-specific CD4 T-cell responses in untreated children mainly consist of IFN-γ-monoproducing cells showing reduced polyfunctionality with a predominant loss of IL-2 responses reflecting the characteristic cytokine profile of effector T cells [23, 28]. After viral suppression by 1 year of ART, the HIV-specific CD4 response shifted towards central memory phenotype in association with increased polyfunctionality and proliferative capacity, the representative features of this memory subset [34]. These observations are consistent with a contraction of the effector response and formation of a persistent central memory compartment as proposed in models of memory T-cell development after antigen removal [35]. Of note, the changes we observed for the HIV-specific CD4 T-cell responses in this study are consistent with previous reports [21, 33] but were more pronounced than previously reported for adults [36]. Children infected with HIV are therefore an interesting patient population in regards to potential immunotherapeutic interventions with the goal of inducing potent T-cell responses to achieve a functional cure after treatment interruption [37].

Globally, TB is the leading cause of death among people living with HIV, and the risk to develop active TB remains severalfold higher in HIV-infected patients, even despite successful ART with full recovery of CD4 counts [38]. Although clear immune correlates of protection remain elusive, there is compelling evidence for a fundamental role of T-cell responses in the successful immune control of TB infection [39]. *Mycobacterium tuberculosis*-specific CD4 T cells from patients with active TB disease exhibit reduced polyfunctionality [40], terminally differentiated memory phenotypes [41], impaired proliferative capacity [40], and higher levels of PD-1 expression [42] compared with patients without active TB disease. In HIV-infected patients, TB-specific CD4 T cells are depleted early after infection [5] and show functional alterations with decreased polyfunctionality and proliferative capacity [8, 12]. Consistent with previous reports in adults [6, 12–14], we only observed modest changes in cytokine profiles of PPD-specific T cells in children on ART with persistently decreased frequencies of polyfunctional CD4 T cells compared with HIV-uninfected children. However, the role of polyfunctional TB-specific CD4 T-cell responses in TB disease remains highly controversial (reviewed in [39]).

A significant cofactor in HIV disease with an influence on immune activation and dysregulation is CMV coinfection, in particular in sub-Saharan Africa, where almost all children acquire CMV early in life [43]. The frequency and function of CMV-specific CD4 T cells in HIV-infected hosts are diminished and only partially restored upon ART [10, 15], which is of particular relevance because, in addition to direct antiviral activity, CMV-specific CD4 T-cell responses are needed to sustain effective CD8 T-cell responses [44]. In our study, similar to the responses against TB, the change in the absolute magnitude of the CD4 response against CMV was strongly correlated to a decrease of PD-1 expression.

The immunoregulatory molecule PD-1 has been identified as a major factor in the complex network of molecular events that results in immune exhaustion in HIV infection [2]. Likewise, in CMV infection, increased PD-1 expression on T cells results in functional exhaustion with diminished cytokine responses and decreased proliferative capacity [45, 46]. Recent studies also highlighted the role of the PD-1 pathway in TB disease, showing increased expression levels of PD-1 in patients with active TB that was correlated with mycobacterial load [47] and decreased TB-specific T-cell responses [42]. In our study, the changes in magnitude of CMV- and TB-specific CD4 T-cell responses on ART was strongly correlated with a reduction of PD-1 expression on effector memory CD4 T cells. Although, in this study, we did not measure PD-1 expression on antigen-specific cells, recent studies have shown that PD-1 expression is increased on TB-specific CD4 T cells from patients with active TB [47] and that blockade of the PD-1/PD-L pathway can recover cytokine and proliferative responses of these cells in vitro [42, 48]. In vitro antagonism of the PD-1/PD-L pathway was also shown to restore HIV- [49] and CMV-specific T-cell responses [45, 46], pointing at new avenues for immunotherapy targeting the PD-1 pathway. However, modulation of the PD-1 pathway in patients with infectious diseases will need to be assessed carefully, as demonstrated by studies of PD-1 knockout mice that show increased pathology during MTB infection exacerbated by vigorous T-cell responses [50].

Our study has a number of important limitations. Due to the observational nature of the study, we can only report on correlations between our findings. The number of cases in our study is relatively small, and our findings should be confirmed in larger cohorts. The follow-up period of this study was short considering the current prospects of lifelong ART for this population, and it calls for long-term follow-up studies, especially with a focus on immune activation for which we only observed little short-term impact of ART. The changes we observed in T-cell responses on ART were more pronounced in the CD4 T-cell compartment. Unfortunately, a more detailed study of CD4 responses is currently constrained by the limited availability of reliable major histocompatibility complex class II tetramers, which would allow identification of antigen-specific CD4 T cells independent of cytokine responses upon in vitro stimulation to allow unbiased characterization of these cells including measurements of PD-1 expression on tetramer-positive populations. Finally, this study was only limited to examine reconstitution of T-cell responses. Future studies should investigate other aspects of the immune system including humoral and innate immunity.

## CONCLUSIONS

In summary, we demonstrate that children have great potential for reconstitution of HIV-specific and copathogen-specific immune responses that are associated with shifts in memory phenotype and PD-1 expression. Immune checkpoint inhibitors that target the PD-1 pathway may represent a potential intervention in patients with insufficient immune reconstitution on ART.

## Supplementary Data

Supplementary materials are available at *The Journal of Infectious Diseases* online. Consisting of data provided by the authors to benefit the reader, the posted materials are not copyedited and are the sole responsibility of the authors, so questions or comments should be addressed to the corresponding author.

## Supplementary Material

Supplementary Figure 1

Supplementary Figure 2

Supplementary Figure 3

Supplementary Figure 4

Supplementary Figure 5

Supplementary Figure 6

Supplementary Figure 7

Supplementary Figure 8

Supplementary Table 1

Supplementary Table 2

Supplementary Material

## Notes

***Acknowledgments.* **We thank all children and their care givers for their time and commitment to this study. We also thank all staff at the Ithembalabantu Clinic (Umlazi, Durban) for their essential support and all core staff members at the HIV Pathogenesis Programme who helped with patient recruitment and sample processing.

***Financial support.* **This work was funded by a grant from the Wellcome Trust (WT104748MA; to P. J. R. G.).

***Potential conflicts of interest.* **All authors: No reported conflicts of interest. All authors have submitted the ICMJE Form for Disclosure of Potential Conflicts of Interest.
