# Supplementary figures and images for: Differential Pathogen-Specific Immune Reconstitution in Antiretroviral Therapy-Treated Human Immunodeficiency Virus-Infected Children
Source: J Infect Dis. 2019 Jan 8;219(9):1407–17. doi: 10.1093/infdis/jiy668 (PMC6467189; doi:10.1093/infdis/jiy668)

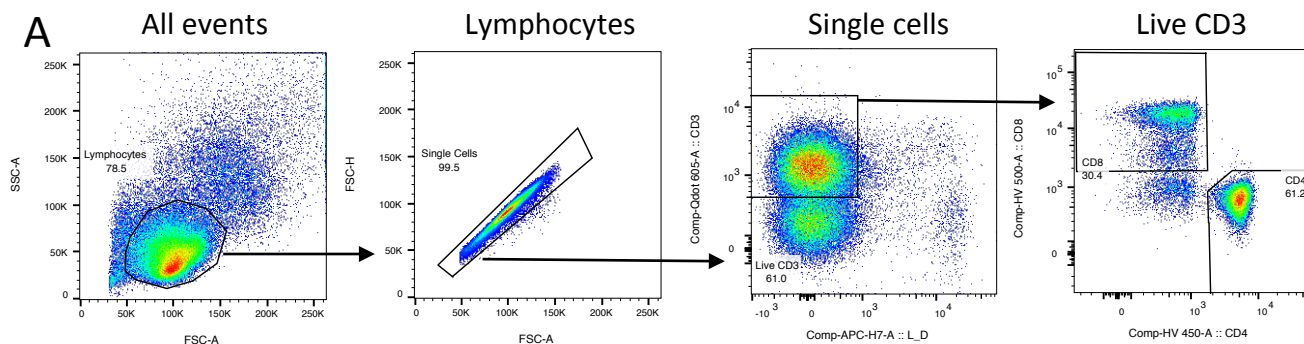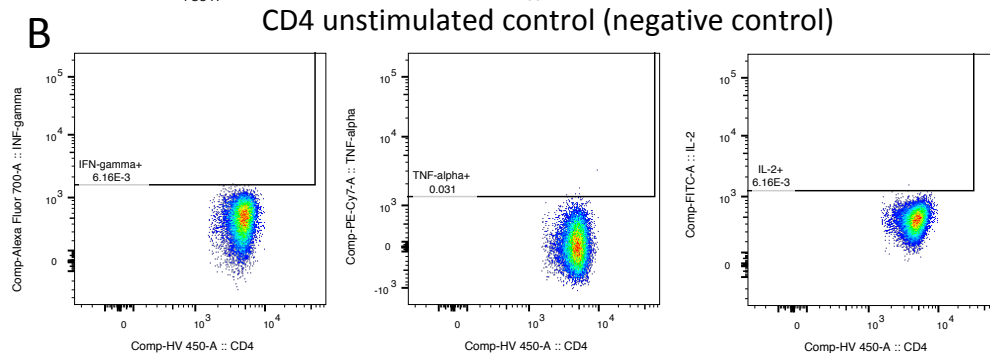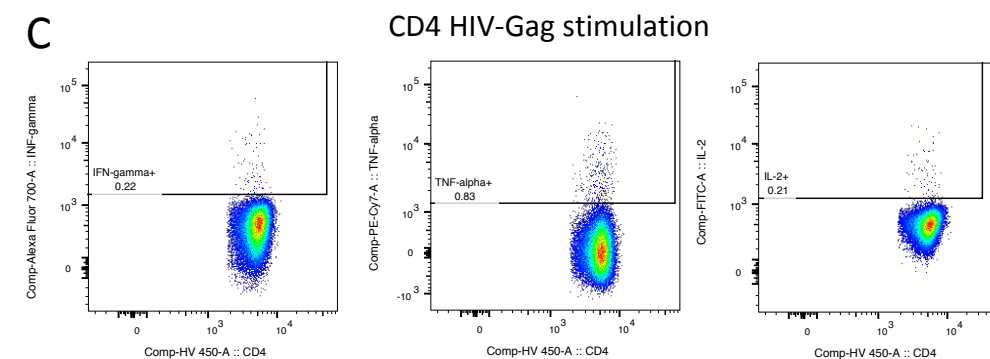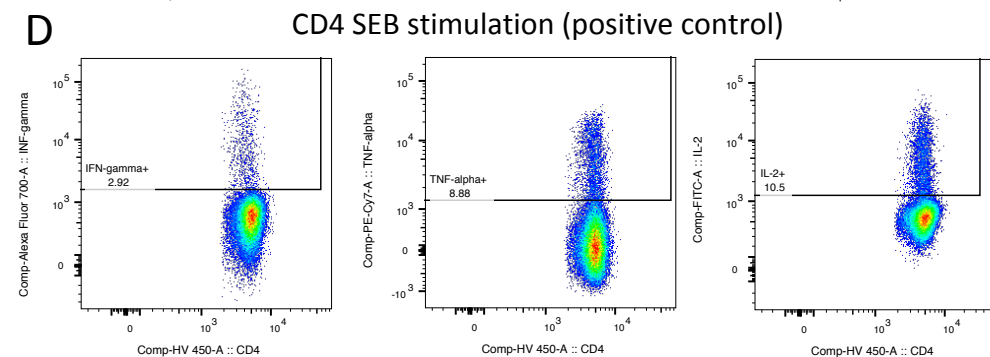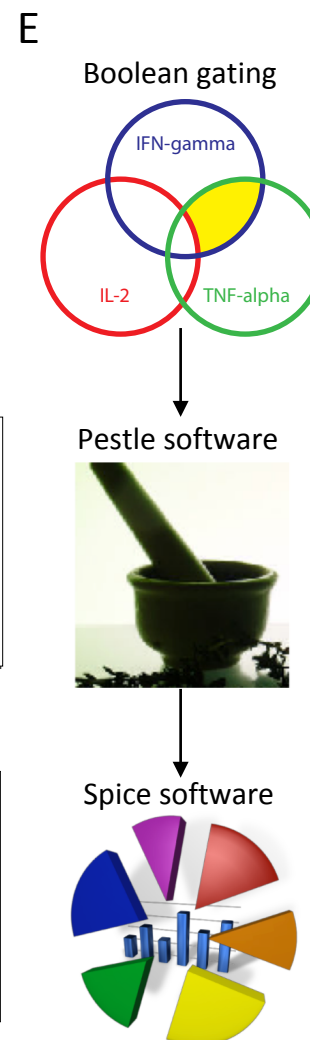

Supplement: Supplementary Figure 1 [file jiy668_suppl_supplementary_figure-1.pdf]

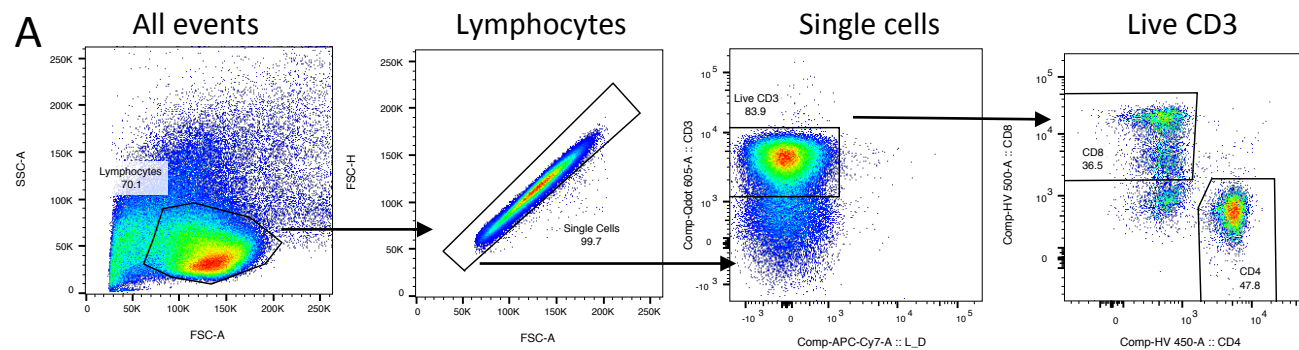

**B**      CD4 gating for cytokine responses

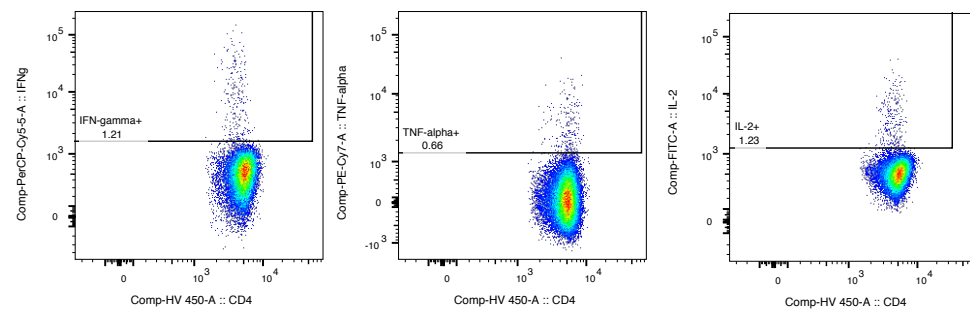

**C**      CD4 gating for memory markers

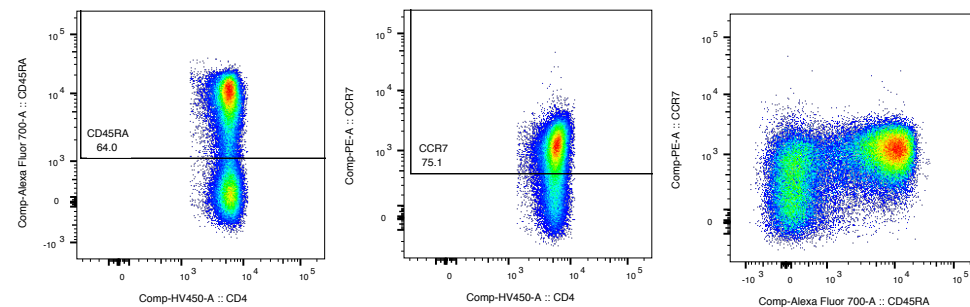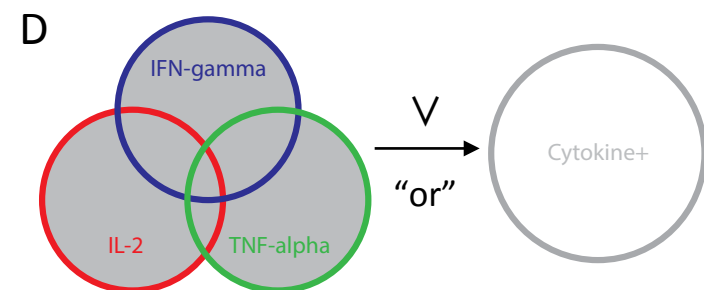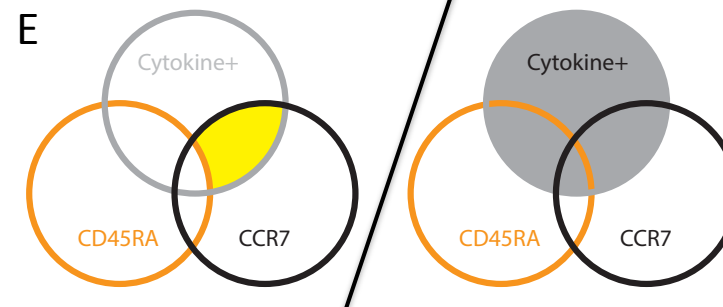

Supplement: Supplementary Figure 3 [file jiy668_suppl_supplementary_figure-3.pdf]

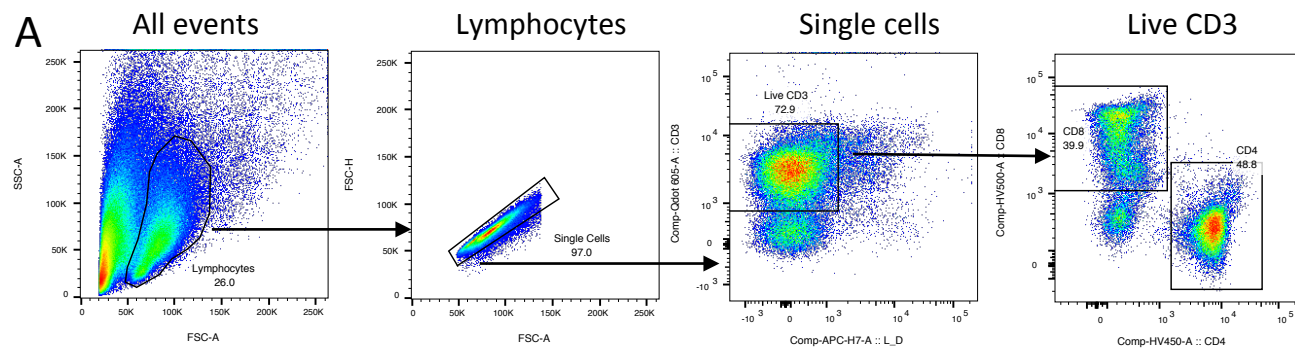

**B** CD4 gating for proliferative responses

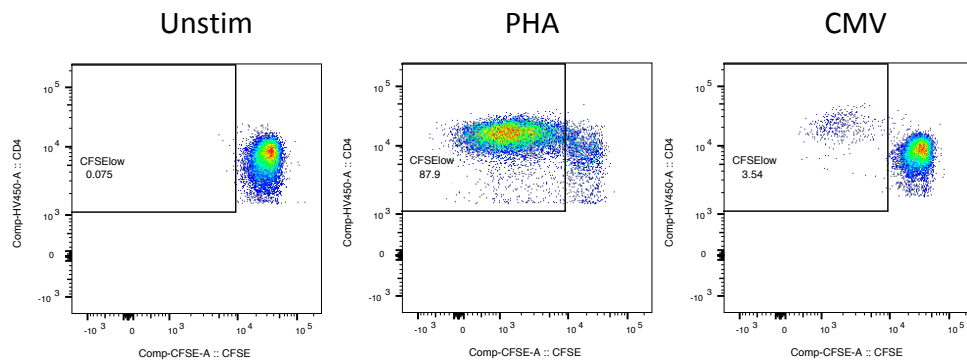

Supplement: Supplementary Figure 4 [file jiy668_suppl_supplementary_figure-4.pdf]

**A**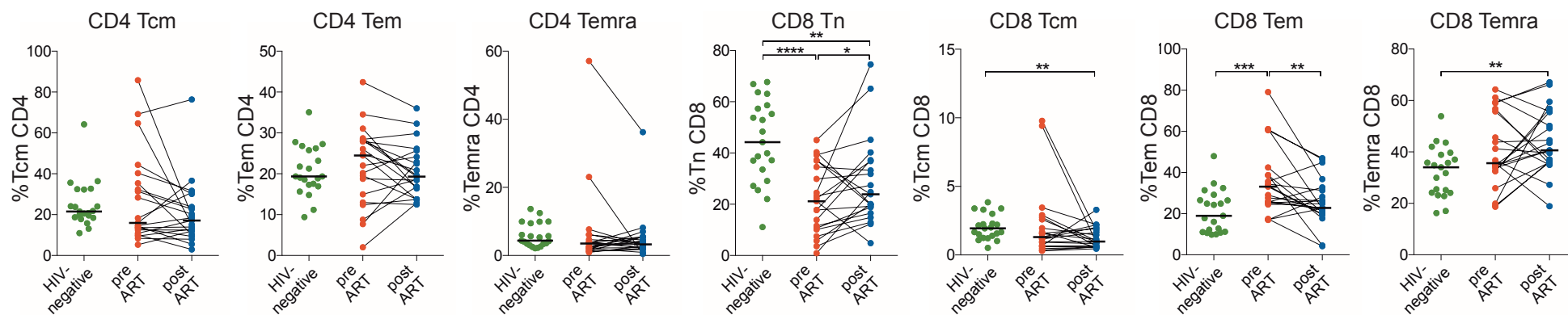**B**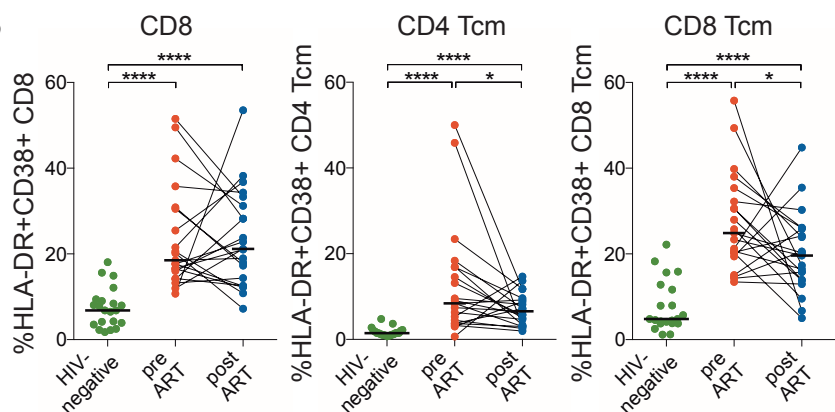**C**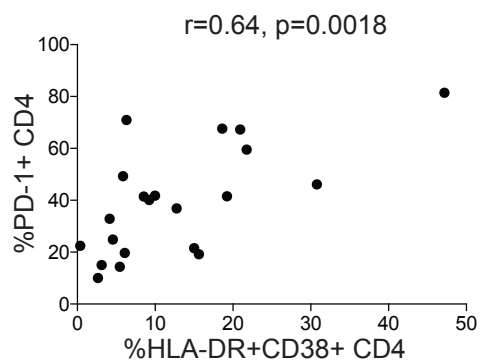**D**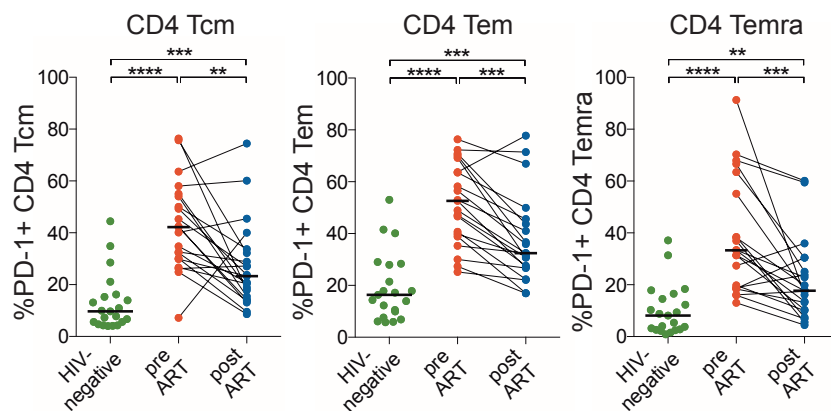**E**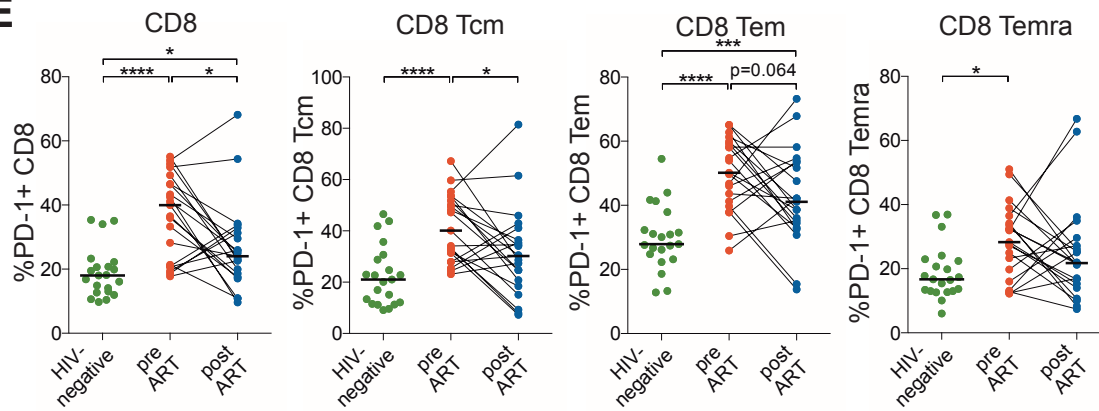

Supplement: Supplementary Figure 5 [file jiy668_suppl_supplementary_figure-5.pdf]

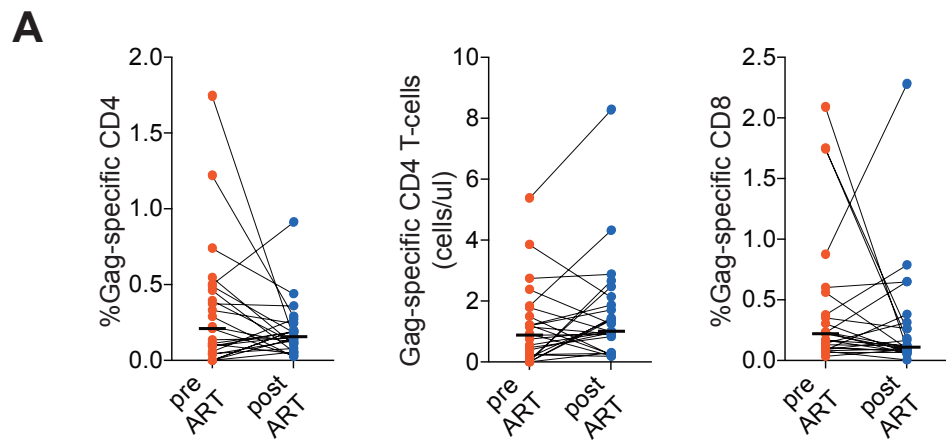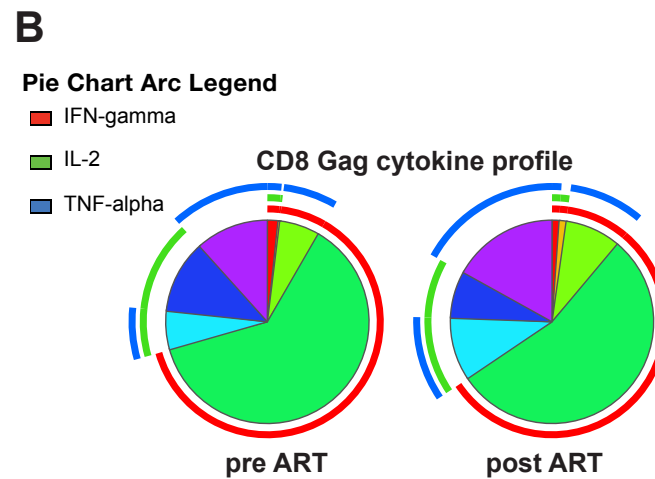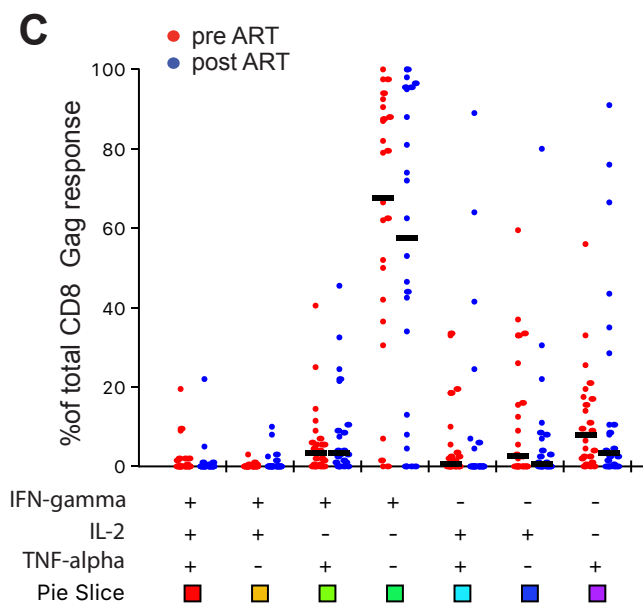

Supplement: Supplementary Figure 6 [file jiy668_suppl_supplementary_figure-6.pdf]

**A****Pie Chart Arc Legend**

IFN-gamma

IL-2

TNF-alpha

**CD8 PPD cytokine profile**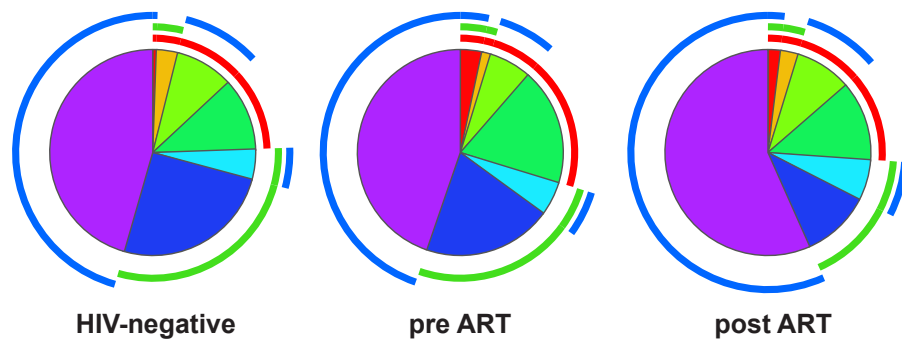**B**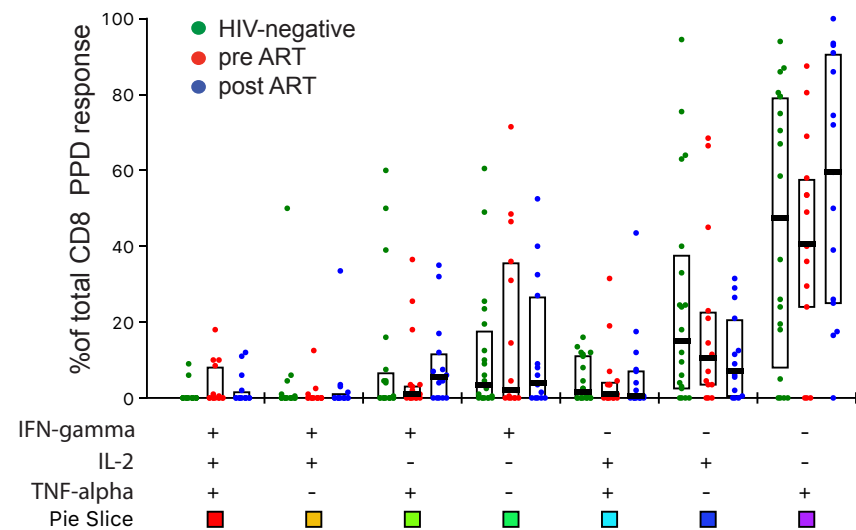**C**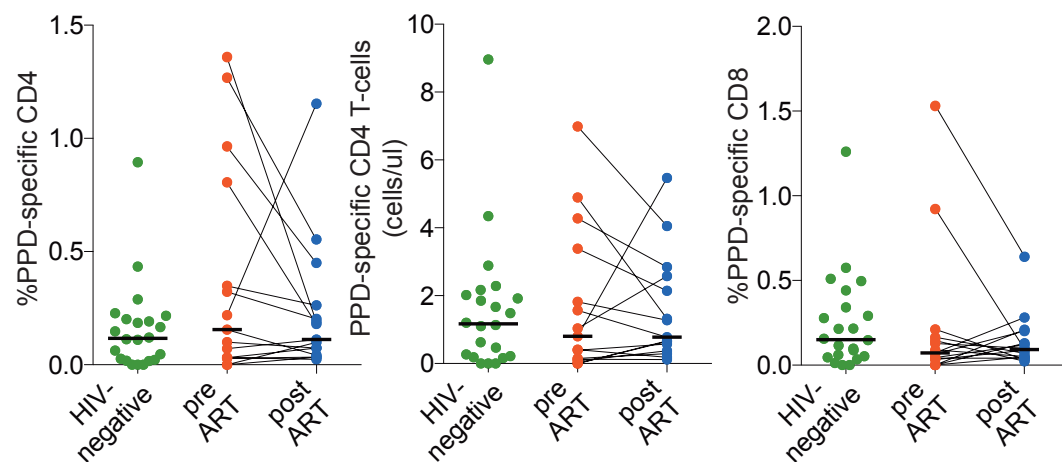**D** $r=0.85, p=0.0061$ 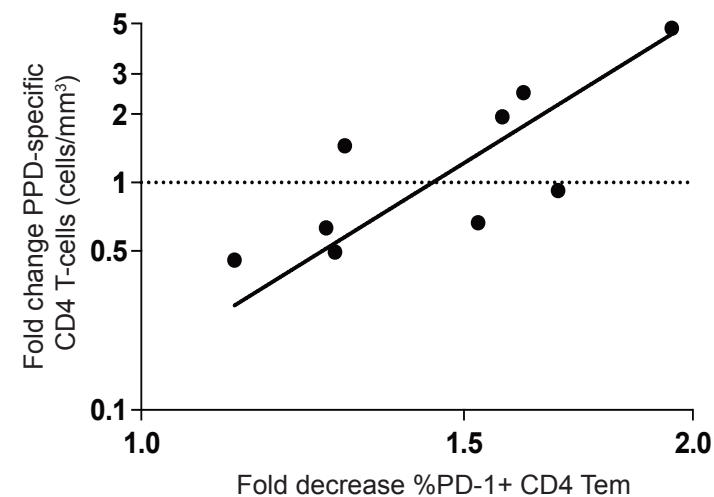

Supplement: Supplementary Figure 7 [file jiy668_suppl_supplementary_figure-7.pdf]

**A**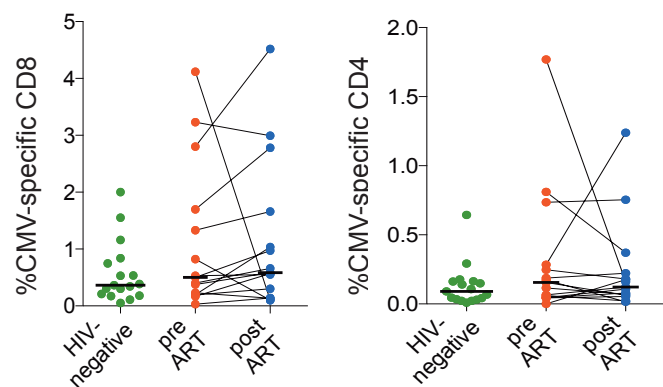**B****Pie Chart Arc Legend**

IFN-gamma

IL-2

TNF-alpha

**CD8 CMV cytokine profile**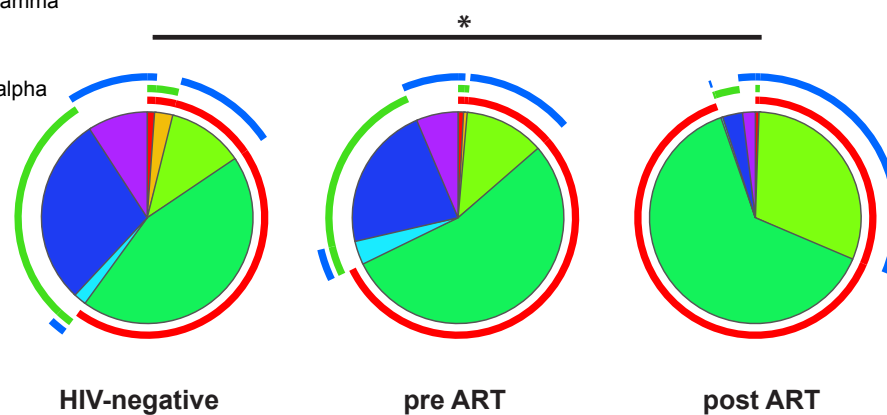**C**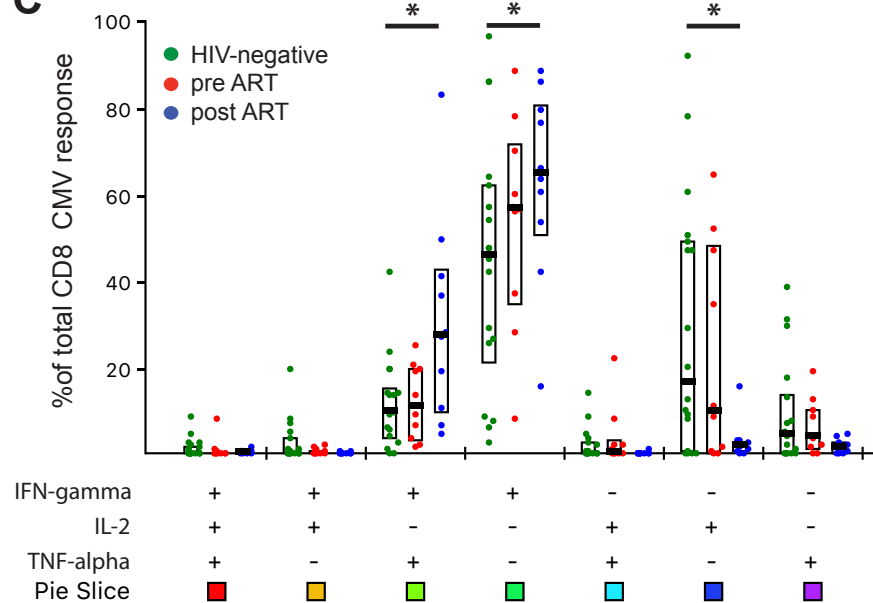**D**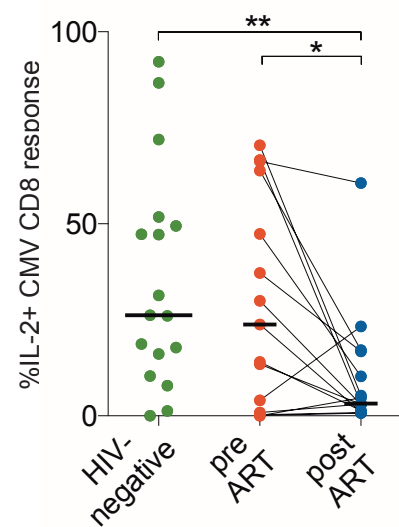

Supplement: Supplementary Figure 8 [file jiy668_suppl_supplementary_figure-8.pdf]
