# Supplementary material for: Differential Pathogen-Specific Immune Reconstitution in Antiretroviral Therapy-Treated Human Immunodeficiency Virus-Infected Children
Source: J Infect Dis. 2019 Jan 8;219(9):1407–17. doi: 10.1093/infdis/jiy668 (PMC6467189; doi:10.1093/infdis/jiy668)
Supplement: Supplementary Figure 2 [file jiy668_suppl_supplementary_figure-2.pdf]

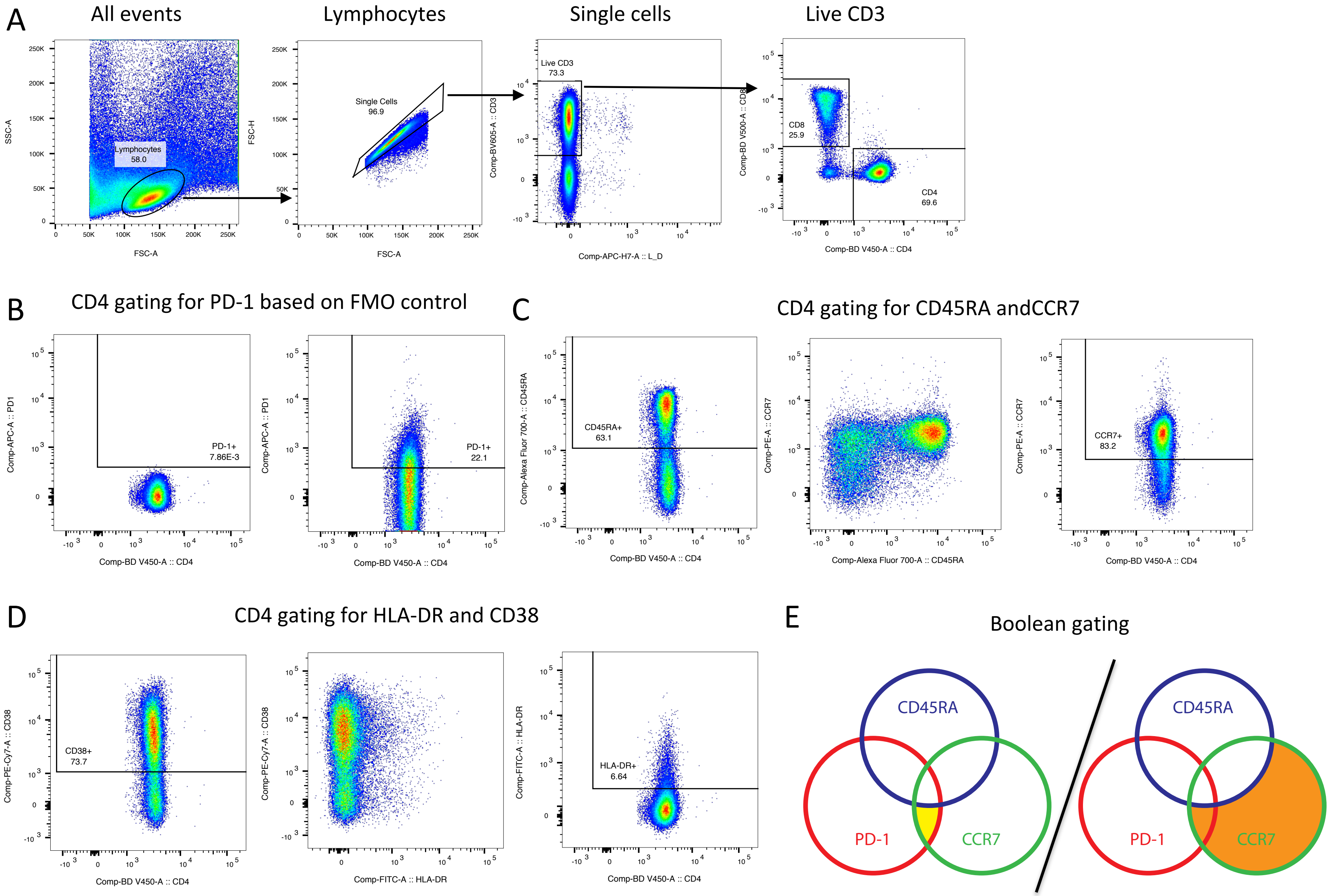
A
All events
Lymphocytes
Single cells
Live CD3
Lymphocytes
58.0
SSC-A
FSC-A
Single Cells
96.9
FSC-H
Live CD3
73.3
Comp-BV605-A :: CD3
Comp-APC-H7-A :: L_D
CD8
25.9
CD4
69.6
Comp-BD V500-A :: CD8
Comp-BD V450-A :: CD4
B
CD4 gating for PD-1 based on FMO control
PD-1+
7.86E-3
PD-1+
22.1
Comp-APC-A :: PD1
Comp-BD V450-A :: CD4
C
CD4 gating for CD45RA andCCR7
CD45RA+
63.1
Comp-Alexa Fluor 700-A :: CD45RA
Comp-PE-A :: CCR7
CCR7+
83.2
D
CD4 gating for HLA-DR and CD38
CD38+
73.7
Comp-PE-Cy7-A :: CD38
Comp-FITC-A :: HLA-DR
HLA-DR+
6.64
E
Boolean gating
CD45RA
PD-1
CCR7
