# Supplementary material for: Differential Pathogen-Specific Immune Reconstitution in Antiretroviral Therapy-Treated Human Immunodeficiency Virus-Infected Children
Source: J Infect Dis. 2019 Jan 8;219(9):1407–17. doi: 10.1093/infdis/jiy668 (PMC6467189; doi:10.1093/infdis/jiy668)
Supplement: Supplementary Table 1 [file jiy668_suppl_supplementary_table-1.docx]

| Panel memory differentiation/activation/PD-1 (n=21, directly after PBMC isolation without cryopreservation) | | | |
| --- | --- | --- | --- |
| Antibody | Flurochrome | clone | company |
| CD3 | BV605 | OKT3 | BioLegend |
| CD4 | BV510 | L200 | BD |
| CD8 | BV421 | RPA-T8 | BD |
| CD38 | PE-Cy7 | HIT2 | BD |
| CCR7 | PE | #150503 | R&D systems |
| HLA-DR | FITC | L243 | BD |
| CD45RA | AlexaFluor700 | HI100 | BioLegend |
| PD-1 | APC | MIH4 | eBioscience |
| Live/dead | Near-IR | N/A | Invitrogen |
|  |  |  |  |
| Panel intracellular cytokine staining (n=25, directly after PBMC isolation without cryopreservation) | | |  |
| Antibody | Flurochrome | clone | company |
| CD3 | BV605 | OKT3 | BioLegend |
| CD4 | BV510 | L200 | BD |
| CD8 | BV421 | RPA-T8 | BD |
| TNF-alpha | PE-Cy7 | MAb11 | BD |
| IL-2 | FITC | 5344.111 | BD |
| IFN-gamma | AlexaFluor700 | B27 | BD |
| Live/dead | Near-IR | N/A | Invitrogen |
|  |  |  |  |
| Panel memory differentiation of cytokine responding cells (n=11, from cryopreserved samples) | | | |
| Antibody | Flurochrome | clone | company |
| CD3 | BV605 | OKT3 | BioLegend |
| CD4 | BV510 | L200 | BD |
| CD8 | BV421 | RPA-T8 | BD |
| TNF-alpha | PE-Cy7 | MAb11 | BD |
| IL-2 | FITC | 5344.111 | BD |
| IFN-gamma | PerCP/Cy5.5 | 4S.B3 | BioLegend |
| CD45RA | AlexaFluor700 | HI100 | BioLegend |
| CCR7 | PE | #150503 | R&D systems |
| Live/dead | Near-IR | N/A | Invitrogen |

| Panel CFSE proliferation assay (n=9) | | |  |
| --- | --- | --- | --- |
| Antibody | Flurochrome | clone | company |
| CD3 | BV605 | OKT3 | BioLegend |
| CD4 | BV510 | L200 | BD |
| CD8 | BV421 | RPA-T8 | BD |
| N/A | CFSE | N/A | Invitrogen |
| Live/dead | Near-IR | N/A | Invitrogen |
