# Supplementary material for: Differential Pathogen-Specific Immune Reconstitution in Antiretroviral Therapy-Treated Human Immunodeficiency Virus-Infected Children
Source: J Infect Dis. 2019 Jan 8;219(9):1407–17. doi: 10.1093/infdis/jiy668 (PMC6467189; doi:10.1093/infdis/jiy668)
Supplement: Supplementary Table 2 [file jiy668_suppl_supplementary_table-2.pdf]

Clinical characteristics of study participants

			Pre-ART				Post-ART		
Participant Identification	Sex	ART	Age (years)	CD4 count (cells/ul)	CD4%	Viral load (copies/ml)	CD4 count (cells/ul)	CD4%	Viral load (copies/ml)
203-33-0004-1	f	ABC/3TC/EFV	12.9	521	31	490000	486	34	<20
203-33-0015-1	f	ABC/3TC/EFV	4.2	550	28	17000	732	44	<20
203-33-0037-1	f	ABC/3TC/EFV	9.2	360	12	7100	696	22	<20
205-33-0001-1	f	ABC+3TC+EFV	4.2	1171	26	48500	1849	38	<20
205-33-0012-1	m	ABC+3TC+EFV	1.4	172	11	17596	1907	32	<20
205-33-0015-1	m	ABC+3TC+EFV	7.2	35	2	25910	692	16.3	<20
205-33-0024-1	m	ABC+3TC+EFV	3.5	138	6	946297	943	27	<20
205-33-0027-1	f	ABC/3TC/LPVr	1.9	1655	29	589931	2185	41.8	<20
205-33-0029-1	m	TDF/FTC/EFV	7.3	60	5	6800	161	9	<20
205-33-0030-1	m	ABC/3TC/EFV	8.8	263	17	140000	427	28	<20
205-33-0033-1	m	ABC/3TC/EFV	6.1	367	3	7800	474	8	<20
205-33-0038-1	m	ABC/3TC/EFV	4.8	1439	28	490000	2302	40	<20
205-33-0040-1	f	TDF/FTC/EFV	15.2	235	21	52000	507	38	<20
205-33-0051-1	m	ABC/3TC/LPVr	1.4	2915	32	940	1833	34	<20
205-33-0056-1	f	ABC/3TC/EFV	7.2	1075	30	120000	1009	35	<20
205-33-0061-1	m	ABC/3TC/EFV	11.5	251	9	540000	511	15	<20
205-33-0062-1	m	TDF/FTC/EFV	13.7	1051	37	24000	1057	33	<20
205-33-0065-1	f	TDF/FTC/EFV	7.9	195	11	250000	430	19	<20
205-33-0066-2	f	TDF/FTC/EFV	13.1	412	15	270000	503	19	<20
205-33-0067-1	m	ABC/3TC/EFV	7.6	516	15	890000	880	28	<20
205-33-0067-2	f	ABC/3TC/EFV	9.5	443	18	1600000	631	29	<20
205-33-0070-1	f	ABC/3TC/EFV	10.6	405	21	52000	674	37	<20
205-33-0071-1	f	TDF/FTC/EFV	7.6	14	5	150000	180	17	<20
205-33-0073-1	f	TDF/FTC/EFV	14.9	41	3	590000	571	22	<20
205-33-0074-1	f	TDF/FTC/EFV	7	397	18	48000	777	32	<20
Median			7.6	397	17	120000	692	29	
IQR			4.5-11.0	183-800	7.5-28	20798-515000	494-1033	19-36	
