# Supplementary material for: Differential Pathogen-Specific Immune Reconstitution in Antiretroviral Therapy-Treated Human Immunodeficiency Virus-Infected Children
Source: J Infect Dis. 2019 Jan 8;219(9):1407–17. doi: 10.1093/infdis/jiy668 (PMC6467189; doi:10.1093/infdis/jiy668)
Supplement: Supplementary Material [file jiy668_suppl_supplementary_material.docx]

**Supplementary Material to**

**“Differential pathogen-specific immune reconstitution in ART-treated HIV-infected children”**

**Maximilian Muenchhoff^a,b,c,d^*****, Emily Adland^a^, Julia Roider^a,b,e,f^, Henrik Kløverpris^f,g,h^, Alasdair Leslie^f,h^, Stephan Boehm^c,d^, Oliver T Keppler^c,d^, Thumbi Ndung’u^b,f,I,j^, Philip JR Goulder^a,b^**

**Content:**

**Supplementary Table 1:** Reagents and panels used for flowcytometry in this study.

**Supplementary Table 2:** Clinical characteristics of study participants.

**Supplementary Figure 1**: Gating strategy for flowcytometric analysis of cytokine responses.

**Supplementary Figure 2**: Gating strategy for flowcytometric analysis of memory differentiation, activation and PD-1 expression.

**Supplementary Figure 3**: Gating strategy for flowcytometric analysis of the memory phenotype of antigen-specific T-cells.

**Supplementary Figure 4:** Gating strategy for flowcytometric analysis of proliferative responses of T-cells upon *in vitro* stimulation for 7 days.

**Supplementary Figure 5:** Additional data of T-cell activation, exhaustion and memory phenotype before and after ART.

**Supplementary Figure 6:** Additional data of HIV-specific T-cell responses before and after ART.

**Supplementary Figure 7:** Additional data of PPD-specific T-cell responses before and after ART.

**Supplementary Figure 8:** Additional data of CMV-specific T-cell responses before and after ART.
